# Bioadhesive Matrix Tablets Loaded with Lipophilic Nanoparticles as Vehicles for Drugs for Periodontitis Treatment: Development and Characterization

**DOI:** 10.3390/polym11111801

**Published:** 2019-11-02

**Authors:** Denise Murgia, Giuseppe Angellotti, Fabio D’Agostino, Viviana De Caro

**Affiliations:** 1 Dipartimento di Discipline Chirurgiche, Oncologiche e Stomatologiche, Università degli Studi di Palermo, 90127 Palermo, Italy; denise.murgia@unipa.it; 2 Dipartimento di Scienze e Tecnologie Biologiche Chimiche e Farmaceutiche (STEBICEF), Università degli Studi di Palermo, 90123 Palermo, Italy; angellotti.gius@gmail.com; 3 Istituto per lo Studio degli Impatti Antropici e Sostenibilità dell’Ambiente Marino, Consiglio Nazionale delle Ricerche (IAS – CNR), Campobello di Mazara, 91021 Trapani, Italy

**Keywords:** buccal matrix tablets, nanostructured lipid carriers, NLC, metronidazole, curcumin, oral mucosal drug delivery, buccal delivery, hydrophilic sponge, periodontitis, oral disease

## Abstract

Periodontitis treatment is usually focused on the reduction or eradication of periodontal pathogens using antibiotics against anaerobic bacteria, such as metronidazole (MTR). Moreover, recently the correlation between periodontal diseases and overexpression of reactive oxygen species (ROS) led to the introduction of antioxidant biomolecules in therapy. In this work, bioadhesive buccal tablets, consisting of a hydrophilic matrix loaded with metronidazole and lipophilic nanoparticles as a vehicle of curcumin, were developed. Curcumin (CUR)-loaded nanostructured lipid carriers (NLC) were prepared using glycyrrhetic acid, hexadecanol, isopropyl palmitate and Tween^®^80 as a surfactant. As method, homogenization followed by high-frequency sonication was used. After dialysis, CUR-NLC dispersion was evaluated in terms of drug loading (DL, 2.2% w/w) and drug recovery (DR, 88% w/w). NLC, characterized by dynamic light scattering and scanning electron microscopy (SEM), exhibited a spherical shape, an average particle size of 121.6 nm and PDI and PZ values considered optimal for a colloidal nanoparticle dispersion indicating good stability of the system. Subsequently, a hydrophilic sponge was obtained by lyophilization of a gel based on trehalose, Natrosol and PVP-K90, loaded with CUR-NLC and MTR. By compression of the sponge, matrix tablets were obtained and characterized in term of porosity, swelling index, mucoadhesion and drugs release. The ability of the matrix tablets to release CUR and MTR when applied on buccal mucosa and the aptitude of actives to penetrate and/or permeate the tissue were evaluated. The data demonstrate the ability of NLC to promote the penetration of CUR into the lipophilic domains of the mucosal membrane, while MTR can penetrate and permeate the mucosal tissue, where it can perform a loco-regional antibacterial activity. These results strongly support the possibility of using this novel matrix tablet for delivering MTR together with CUR for topical treatment of periodontal diseases.

## 1. Introduction

Bacterial infections are among the most common diseases that could affect the oral mucosae. An immunocompromised state of the oral cavity is related to systemic and local factors that could allow the development of infections. Moreover, antibiotic multi-resistance is becoming frequent in bacterial pathogens and it may cause the overgrowth of microorganisms [1].

In particular, periodontal disease is a bacterial infection associated with the overgrowth of anaerobic organisms and, in certain instances with the micro-aerophilic ones, resulting in chronic inflammation of the gingiva [2]. Their action results in progressive connective tissue loss enhanced by the host’s inflammatory factors, which can lead to tooth loss [3].

In addition to bacterial action, frequently oral lesions are exasperated by the presence of reactive oxygen species (ROS). Their overproduction that is not balanced by an adequate level of antioxidants can prompt oxidative stress damages. In periodontal disease, most of the damage is induced by inflammatory mediators, involving free radical increasing from the neutrophil/macrophage anti-bacterial response [4]. For this reason, the administration of antioxidant substances in periodontal diseases has recently been extensively studied [5].

Periodontitis treatment is usually focused on the reduction or eradication of periodontal pathogens using antibiotic agents [6].

Metronidazole (MTR) is the first-line antimicrobial agent in the treatment of inflamed periodontal pockets [7], attributable to its activity spectrum against both Gram-negative and Gram-positive anaerobic bacteria and its limited side effects [6,8]. Nonetheless, a prolonged systemic administration of MTR in periodontal disease can raise several issues, such as the increased risk of antibiotic resistance and the presence of side effects like nausea, diarrhea and pseudomembranous colitis [9].

Since oxidative stress is involved as an etiological component of these diseases, a treatment involving MTR together with antioxidant agents could be suitable to obtain synergic action.

Curcumin (CUR) is a natural polyphenolic compound obtained from the rhizome of *Curcuma longa*, where it is present with other characteristic compounds (demethoxycurcumin and bisdemethoxycurcumin) known as curcuminoids [10]. It has been studied for its several pharmaceutical properties, such as being antibacterial [11], antioxidant, anti-inflammatory [12], anti-cancer and a wound healing agent [13]. 

This compound was frequently used in the treatment of oral lesions, periodontitis in particular, because of its several mechanisms and molecular targets mediating the anti-inflammatory action. It suppresses the activation of the transcription factor NF-κB, cytokines such as TNF-α, IL-1, -2, -6, -8, -12, mitogen-activated protein kinase (MAPK) and c-Jun N terminal kinase (JNK), and downregulates the expression of cyclo-oxygenase-2 (COX-2), lipoxygenase (LOX) and the inducible nitric oxide synthase (iNOS) [14]. Evidences from both in vitro and in vivo studies show that CUR is able to decrease the response of immune cells to periodontal disease-associated bacterial antigens, inhibiting periodontal tissue destruction [15].

CUR is chemically unstable, practically insoluble in water and soluble in solvents such as ethanol and acetone. In the gastrointestinal environment, it suffers slow solubilization and fast degradation that limit absorption. Moreover, a short plasma half-life drastically reduces its action in vivo [16].

Considering the limits of both MTR and CUR systemic administration and the site-specific condition of destructive periodontal disease, a local administration could be an improved treatment for several oral pathologies, since there are minor risks of systemic side effects [17]. 

Conventional dosage forms may not be accurate due to fast dissolution and disintegration during the application period, while drug delivery systems (DDS) expressly designed to deliver substances in oral mucosa are preferred to promote high concentrations of actives directly into periodontal pockets, improving the in loco treatments. They are designed to stay in contact with the mucosa longer, provide more accurate drug dosing and covering a larger surface area, suited to protect oral wound surfaces [18]. Furthermore, due to its relative immobility, buccal mucosa is a desirable region for retentive systems used for transmucosal drug delivery.

Mucoadhesion is a critical parameter for buccal administration, so materials with optimal adhesive properties, such as mucoadhesive polymers, should be selected. These polymers play an important role in designing bioadhesive oral formulations, since their ability to create strong adhesive contacts with tissues increases the residence time in the target site of such systems [19]. 

Recently, new mucoadhesive systems, suitable to treat buccal affections, were developed. Buccal tablets are one of the most commonly investigated dosage forms for buccal administration, especially the mucoadhesive ones that give less discomfort in drinking and speaking than the conventional tablet. They can promote drug absorption rates by slow dissolution and erosion [20]. Moreover, molecular size affects the permeability of macromolecules through the oral mucosa, so the drug incorporation as part of nanostructured delivery systems could be developed to enhance the penetration on the target site. Thus, bioadhesive nanoparticles could be more advantageous due to their ability to create intimate contact with larger mucosal surface areas [21].

Nanostructure lipid carriers (NLC) represent a new generation of nanoparticles together with solid lipid nanoparticles (SLN). They are colloidal carrier systems able to deliver active substances, and protect them against chemical degradation, and are mainly used for a controlled release in topical administration [22].

The aim of this work is the formulation, development and characterization of buccal tablets obtained from hydrophilic sponges made by NLC with curcumin (CUR-NLC) entrapped in a dry hydrophilic matrix loaded with MTR. The obtained DDS consists of a bifunctional system able to release CUR and MTR once on the application site, being suitable for the local treatment of several oral diseases.

## 2. Materials and Methods 

### 2.1. Materials

Curcumin (CUR) was purchased from Alfa Aesar (Haverhill, MA, USA), 1-Hexadecanol (HEXA) from Farmalabor (Canosa di Puglia, Italy) isopropyl palmitate (IP), glycyrrhetic acid (GA) and metronidazole (MTR) from A.C.E.F S.p.a. (Fiorenzuola d’Arda, Italy), Tween 20 and Polyvinylpyrrolidone (PVP-K90) from Sigma-Aldrich (Milan, Italy), Hydroxyethylcellulose (Natrosol™ 250) from Galeno (Comeana PO, Italy) and trehalose dihydrate from la Hayashibara Shoji Inc (Okayama, Japan).

Non-enzymatic artificial plasma of pH 7.4 (PBS) was prepared dissolving 2.80 g of KH_2_PO_4_ and 20.5 g of Na_2_HPO_4_ in1 L of distilled water and the same conditions were used for the non-enzymatic artificial plasma of pH 7.4 (PBS) with 5% and 10% of DMSO.

A buffer pH 6.8 solution simulating salivary fluid was prepared using KCl (1.5 g), NaHCO_3_ (1.75 g), (KSCN 0.5 g), Na_2_HPO_4_ H_2_O (0.5 g) and lactic acid (1 g) in distilled water; 0.9% saline solution was prepared by dissolving 9 g of NaCl in 11 L of distilled water.

Citrate buffer 10 mM (pH 6.2) used for NLC preparation was obtained dissolving 2.675 g of sodium citrate dehydrate and 0.190 g of citric acid monohydrate in 1 L of distilled water.

All chemicals and solvents of analytical grade were purchased from VWR International (Milan, Italy) and used without further purification. Porcine mucosae were kindly supplied by the Municipal Slaughterhouse of Villabate (Palermo, Italy).

For data processing, we used Kaleidagraph v. 3.5 (Synergy Software Inc, Reading, PA, USA) and Curve Expert 1.34 for Windows as software.

### 2.2. Lipid Ratio Mixtures Screening Studies

Lipid mixtures (LM) prepared changing both the ratio of the components and CUR are reported in Table n.1.

A calculated amount (1 g) of each LM was melted and stirred using a silicon bath placed on a hot plate (Heidolph MR2001 K equipped with EKT 3001 electronic temperature control, Germany), until the mixture appeared clear and CUR was completely solubilized, then cooled at room temperature and finally stored at 2–4 °C. After 24 h, the melting point of the mixtures was evaluated using Büchi Melting Point B-540 instrument sets at increasing temperature speed of 5 °C/min. The analysis was carried out in triplicate and the result was expressed as the mean of the values obtained.

### 2.3. Preparation of CUR-Loaded NLC

Aliquots of 100 mg of each lipid mixture were added to 20 mL of 10 mM citrate buffer of pH 6.2 with 0.5% or 1% (w/v) of Tween 20 as a surfactant and homogenized (Polytron Model PT MR 2100 Homogenizer, Kinematica, Luzern, Switzerland) for 1 min. Then a high-frequency sonication treatment using a SONOPULS instrument (mod. HD 2070, Bandelin, Berlin, Germany) at a pulsating frequency of 20 kHz with 0.7 s of activity (on) and 0.3 s of inactivity (off) was performed. Samples were treated with two cycles of sonication (10 min each), the first one at room temperature and the second at 0 °C by immersing the container in an ice/water/NaCl bath.

### 2.4. NLCs Characterization

#### 2.4.1. Dynamic Light Scattering (DLS) and Z-Potential Measurements

The mean particle size (Z-average), the polydispersity index (PDI) measuring the distribution of the nanoparticle population and the electrical charge on the surface of the nanoparticles (zeta potential, ZP) were determined using a Nano-ZS Zetasizer (Malvern Instruments, Malvern, UK). DLS measures the fluctuations in scattered light intensity due to diffusing particles and represents one of the most powerful techniques to assess particle size [23]. To avoid multiple scatterings of the light caused by a high concentration of particles, samples were prepared by diluting 1 mL of fresh NLC dispersion in 9 mL distilled water. All analyses were performed at 25 °C, under an electrical field of 40 V for ZP analyses, carrying them out in duplicates. Empty NLCs (without CUR) were analyzed to evaluate a potential CUR influence on mean particle size, distribution and stability.

#### 2.4.2. Morphology by Scanning Electron Microscopy (SEM)

SEM analyses were performed to evaluate the morphology and topographic characteristics of both loaded nanoparticles and sponges loaded with MTR and CUR-NLC.

Measurements were carried out using a Zeiss EVO MA10 (Carl Zeiss Microscopy GmbH, Jena, Germany) scanning electron microscope, equipped with a SE-Everhart-Thornley secondary electron detector having as source of electrons a Lanthanum Hexaboride (LaB_6_) cathode. The accelerating voltage and probe voltage were, respectively, 20 keV and 10 pA.

The scanning electron microphotographs were acquired in ultra-vacuum condition (HV, about 10^−7^ mbar) and magnified up to 50.000× (200 nm).

To analyze CUR-NLCs, a few drops of fresh CUR-NLC dispersion put on an aluminium stub were placed in a CaCl_2_ desiccator at 8 °C for 24 h and then coated with an ultrathin layer of gold (thickness about 2 nm) with an AGAR Sputter Coater type system, to increase the surface electrical conductivity. The gold coating was also made for the samples of sponge loaded with MTR and CUR-NLC before each analysis.

#### 2.4.3. Entrapment Efficacy, Drug Loading and Drug Recovery of NLCs

The entrapment efficiency (EE), loading capacity (LC) and Drug Recovery (DR) were indirectly determined, calculating the amount of free CUR in the aqueous phase, applying Equations (1)–(3), respectively [24]:

(1) $$EE\%\left(\frac{w}{w}\right)=\;\frac{\mathrm{CUR}\;\mathrm{in}\;\mathrm{formulation}-\;\mathrm{free}\;\mathrm{CUR}\;}{\mathrm{CUR}\;\mathrm{in}\;\mathrm{formulation}}\times 100,$$

(2) $$DL\%\left(\frac{w}{w}\right)=\;\frac{\mathrm{CUR}\;\mathrm{in}\;\mathrm{formulation}-\;\mathrm{free}\;\mathrm{CUR}\;}{\mathrm{Lipids}\;\mathrm{amount}\;}\times 100,$$

(3) $$DR\%\left(\frac{w}{w}\right)=\;\frac{\mathrm{CUR}\;\mathrm{in}\;\mathrm{formulation}\;}{\mathrm{CUR}\;\mathrm{used}}\times 100.$$

##### CUR Quantification in Fresh NLC Dispersion

At the end of the NLCs preparation process the volume of the whole formulation was measured. A total of 200 μL of NLC dispersion was withdrawn, transferred into a 10 mL flask and brought to volume with methanol to solubilize all components. Samples were analyzed by HPLC to quantify the total CUR (free and encapsulated) and detect any degradation products [25].

The free CUR, non-entrapped in nanoparticles, was investigated by two methods:

- *Dialysis assay:* Dialysis tube (molecular weight cut off, MWCO, 12–14,000 Da, Visking Dialysis Membrane, Medicell Membranes Ltd., London, UK) was pre-activated and filled by 2 mL of CUR-NLC dispersion and submerged in 350 mL of distilled water, keeping at room temperature and under magnetic stir. After 24 h both the dispersion inside the tube and the external water were analyzed by HPLC.
- *Ultrafiltration assay:* Aliquots of 0.45 mL of fresh samples were centrifuged (Microfuge 22R, Beckman coulter™ Brea, CA, USA) in two Ultrafree-MC (Millipore, Burlington, MA, USA) devices, with membrane cut-off of 10,000 NMWL and 30,000 NMWL, at 8000 rpm and 4 °C for 30 min [26]. In the end, the liquid ultrafiltrate was analyzed by HPLC.

### 2.5. Preparation of Sponges Loaded with MTR and CUR-NLC.

Firstly, a gel made by trehalose (30%), PVP K90 (0.8%) and Natrosol (5%) was prepared by adding the components to distilled water at 60 °C and keeping in an ultrasonic bath until it appeared dense, clear and completely homogeneous. Afterwards, it was stored at 4 °C for 24 h before use.

Sponges were prepared by adding in two different moments, 100 mg MTR and 4 g of gel to 20 mL of CUR-NLC dispersion. After both the addictions, the sample was gently mixed and then treated with a high-frequency sonication (20 kHz) with cycles of 0.7 s (on) and 0.3 s (off) for 2 min, keeping an ice/water/NaCl bath around it. At the end the sample was stored at −80 °C for a night and then moved in a freeze-dryer (Labconco FreeZone^®^ 2.5 Liter Freeze Dry System) with a 0.014 mPa vacuum for 24 h.

The amount of MTR and CUR loaded in the sponges were determined analyzing three samples of each lyophilized preparation in methanol, by UV-Vis spectrophotometry. Therefore, the morphology of the obtained matrix was evaluated by SEM analysis.

#### Tablets Preparation

Two steel tablet moulds (PerkinElmer IR Accessory, Waltham, MA, USA) were used to have tablets with different dimensions and weight. Sponges of 20 mg (to obtain Tablet A) and 200 mg (to obtain Tablet B) were placed in molds with a d = 0.47 cm or d = 1.3 cm, respectively. Both were compressed by a 300 g weight for about 30 min. The results are reported as average ± SE (*n* = 20).

### 2.6. Tablets Characterization

#### 2.6.1. Porosity

Using freeze-drying the solvent is removed, and the space originally occupied by the solvent is retained for the porous structure. The tablet porosity was calculated mathematically, as total pore volume, using the following equation [27]:

$$\%Porosity=\frac{pratical\;volume-theoritical\;volume}{pratical\;volume}\times 100.$$

The bulk volume of the total ingredients of one sponge was calculated according to the equation:

$$Theoritical\;volume=\sum \frac{m_{n}}{\rho_{n}}$$

where *m* and ρ are the mass and density of each ingredient, respectively.

The true volume of tablets was obtained calculating their dimensions as cylinders:

$$Practical\;volume=\pi r^{2}\times h.$$

The results of these measurements were also supported by the SEM scanning electron micrographs.

#### 2.6.2. Swelling Test

Swelling tests were performed on B tablets with both weight and optic assessments. For weight tests, tablets were placed on microscope slides and weighted by an analytic balance (Mod. AE 240, Mettler-Toledo S.p.A., Milan, Italy). Every 5 min for 20 min, 0.5 mL of artificial saliva (pH 6.8) were added on the tables and after the removal of the excess water with a filter paper, the weight was assessed. The water uptake was quantified gravimetrically until the weight-plateau or the cleavage of the tablets. Tests were performed on six different tablets and results were reported as means ± SE (*n* = 9; *p* < 0.05).

$$\mathrm{Swelling}\;\mathrm{Index}=\frac{W_{0}+\left(W_{t}-W_{0}\right)}{W_{0}}\times 100,$$

where *W_t_* is the weight of film at time *t*, and *W*_0_ is the weight of dry film.

Both plan and frontal optical assessments were performed. Samples placed on glass were positioned on graph paper and wet every 5 min for 2 h by 0.5 mL of simulated saliva (pH 6.8, 37 °C). Each time interval a photograph was taken to evaluate any change in the tablet’s morphology.

#### 2.6.3. Ex Vivo Mucoadhesion Strength Measurement

The ex-vivo mucoadhesion strength tests were performed on Tablet B using the modified two armed physical balance method [28]. As model tissue, porcine buccal mucosa excised from slaughtered pigs was used without any pre-treatment. A cyanoacrylate resin (Super Attak Loctite^®^, Henkel Italia Srl, Milan, Italy) was used to glue a piece of mucosa on a glass support kept in a vessel placed in a thermostatic bath at 37 ± 1 °C. This temperature was maintained during the whole experiment. Before starting the measurements, the mucosa was wetted with 50 µL of simulated salivary fluid. The tablet was fixed using double-sided tape to the lower side of a rubber stopper hanging from the balance arm and then placed just touch the wet mucosal surface; a light force with a fingertip was applied for 20 s.

The measures started 5, 10, 15 and 20 min after tablet placement. The grams required to detach it from the mucosal surface provided the measurement of mucoadhesive strength.

The force of adhesion and the detachment force were calculated using the following equations, respectively [28], and each experiment was performed in triplicate:

Force of adhesion (N)= g × 9.81/1000,

$$Detachment\;force\;\left(N/m^{2}\right)=\;Force\;of\;adhesion\;\left(N\right)/surface\;area\;\left(m^{2}\right).$$

#### 2.6.4. In Vitro CUR and MTR Release Studies

The in vitro CUR and MTR release from tablets was carried out using a Franz diffusion cell (Permeagear, flat flange joint, 9 mm orifice diameter, 15 mL acceptor volume, SES GmbH-Analysesysteme, Bechenheim, Germany). A Cellulose nitrate membrane with a 0.45 µm pore size (Millipore) was soaked with acceptor fluid and fixed between the donor and receptor compartment [29].

The receiver chamber was filled with phosphate buffered to pH 6.2 plus Tween 20 (8% w/v) as a surfactant to increase the solubility and stability of MTR and CUR in the receiver media [30].

The temperature of receptor media was kept at 37 ± 0.5 °C. The donor chamber was filled with Tablet A (20 mg, area = 0.2 cm^2^) containing MTR and CUR-NLC and 0.5mL of simulated saliva. At regular time intervals (15 min), samples (0.5 mL) were withdrawn from the acceptor compartment and the sample volume was taken out and replaced with fresh fluid. The samples were analysed by UV-Vis using the appropriate blank and calibration curve. The permeation experiments were carried out for 3 h and results were reported as means ± SE (*n* = 6). Release data were elaborated using Kaleidagraph v. 3.5 software and fitted to the semi-empirical equation that is usually applied to describe drug release from polymeric systems [31]. Linear or non-linear least squares fitting methods were used to determine the optimum values for the parameters present in each equation. Fittings were validated by using χ^2^. A *p*-value of less than 0.05 was considered to be statistically significant.

### 2.7. Ex Vivo Permeation and Penetration of CUR and MTR throughout Porcine Buccal Mucosa

The permeation or/and penetration of CUR and MTR released from tablets through the porcine buccal mucosa, was evaluated using Franz type diffusion cells (Permeagear, flat flange joint, 9 mm orifice diameter, 15 mL acceptor volume, SES GmbH-Analysesysteme, Bechenheim, Germany), used as a two-compartment open model. Mucosal specimens (kindly supplying by Municipal Slaughterhouse of Villabate, Palermo, Italy) consisted of tissue removed from the vestibular area of the retromolar trigone (buccal mucosa) of freshly slaughtered domestic 6–8-month-old pigs were used as a membrane. They were prepared as described previously [32].

The specimens, transferred in the laboratory within 2 h from animal sacrifices, were surgically treated to remove the excess of adipose and connective tissues and then stored at –20 °C for periods up to one week. Before the experiments they were equilibrated at room temperature and dipped for about 1 min in saline solution at 60 °C; the connective tissue was carefully peeled off from the mucosa (slides 250 ± 25 μm and 100 ± 25 μm thick for buccal and sublingual tissues, respectively) to obtain the heat-separated epithelium along with the intact basal lamina [33].

The thickness was measured using a digital micrometer. After this heath treatment, which is able to not modify the permeability and/or integrity of the mucosae [32], specimens were equilibrated in PBS for about 3 h at room temperature to remove biological matter that could interfere with analysis. The equilibration medium was replaced with fresh PBS every 15 min.

The Franz diffusion cells were equilibrated for 30 min at 37 ± 0.5 °C, mounted with buccal mucosa as membrane, PBS (pH 7.4) with 10% DMSO as acceptor compartment and 1 mL and simulated saliva in donor chamber. This step was followed by the removal of donor compartment solution, immediately replaced with a Tablet A (area = 0.2 cm^2^) containing MTR and CUR-NLC and 0.4mL of simulated saliva. At regular time intervals (30 min), samples (0.5 mL) were withdrawn from the acceptor compartment and the sample volume was taken out and replaced with fresh fluid. The permeation experiments were carried out for 4 h. The CUR and MTR amount in acceptor chamber were quantitatively determined by UV-Vis spectrophotometry using the appropriate blank and calibration curve and results were reported as means ± SE (*n* = 12).

At the end of each experiment, mucosal integrity was checked as previously described [34].The flux values (*Js*) across the membranes were calculated at the steady state per unit area by linear regression analysis of permeation data, following the relationship *Js* = Q/At (mg/cm^2^·h), where Q is the amount of drugs recovered in the acceptor compartment, A is the cross-sectional area available for diffusion (0.636 cm^2^), and t is the time of exposure (h). Data were elaborated using Kaleidagraph 3.5 (Sinergy Software Inc., Reading, PA, USA) as software.

#### Quantification of CUR and MTR Entrapped into Porcine Mucosa

At the end of each experiment, the residual MTR and CUR amount entrapped into the mucosal tissue were quantified by extraction. Each mucosa specimen was washed with PBS (3 × 2 mL) and was then dipped for 5 min in warmed (50 °C) methanol (1.5 mL). The extraction was repeated three times and the collected mother liquors were quantitatively transferred in a 5 mL flask and brought to volume. The amount of drugs extracted was evaluated by UV and HPLC analysis using the appropriate calibration curve and blank. The same extraction treatment was also performed on mucosal specimens subjected to an experimental phase in the absence of drugs and was used as the control. Data were reported as percentage amount of CUR and MTR into the tissue. Moreover, the Accumulation (Ac) parameter was calculated using the following equation:

Ac (cm) = [Q (mg)/A (cm^2^)]/Cd (mg/cm^3^)

where Q is the amount of drug which remains entrapped into the buccal tissue at the end of permeation experiments, A is the active cross-sectional area available for diffusion (0.636 cm^2^), and Cd is drug concentration in the donor compartment (mg/cm^3^) [35].

### 2.8. Drugs Assay

#### 2.8.1. UV-Vis Method

The amount of drugs loaded in NLCs and sponges, as well as drugs detected during release and permeation experiments, were measured spectrophotometrically using the appropriate calibration curves and blanks (UV/VIS mod. Pharma Spec 1700, Shimadzu, Tokyo, Japan) with simple, accurate and reproducible methods.

For CUR determination, for λ_max_ = 428 nm, two calibration curve in methanol were performed: In the linearity range of 0.0002–0.0075 mg/mL, the regression equation was Abs = −0.0289 + 132x (mg/mL) (R = 0.999, SE 0.0101) and in the linearity range of 0.0025–0.01 mg/mL, the regression equation was Abs = −0.0108 + 136 x (mg/mL) (R = 0.997, SE 0.0397).

The calibration curve in the phosphate buffer of pH 6.2 plus Tween 20 (8% w/v) was in the linearity range of 0.0001–0.001 mg/mL, and the regression equation was Abs = 0.01277 + 160x (mg/mL) (R = 0.997, SE 0.0105).

For MTR determination were found validation parameters in methanol, in PBS and in phosphate buffer (pH 6.2) plus Tween 20 (8% w/v), at λ_max_ = 310 nm. For methanol, the linearity range was 0.0025–0.03 mg/mL, and the regression equation Abs = 0.0011+ 62x (mg/mL) (R = 0.999, SE 0.0092); for PBS the linearity range was 0.0025–0.0125 mg/mL, and the regression equation Abs = 0.0011 + 61.78x (mg/mL) (R = 0.999, SE 0.0040). For the phosphate buffer (pH 6.2) plus Tween 20 (8% w/v), the linearity range was 0.0025–0.02 mg/mL, and the regression equation was Abs = 0.0771 + 46.8x (mg/mL) (R = 0.998, SE 0.0102).

At the testing concentrations, no interferences between drugs and components of formulations were observed and no change in drug absorbance at its λmax was experienced in the presence of excipients. In analogy, the amount of drugs founded in acceptor and/or entrapped into the membrane were measured after withdrawal or extraction from mucosal tissue by methanol. Intraday and interday variations, observed during the collection of experimental data, were lower than sensibility.

#### 2.8.2. HPLC Method

HPLC analyses were performed with an HPLC Shimadzu LC-10AD VP instrument (Tokyo, Japan) equipped with a binary pump LC-10AD VP, a UV SPD-M20A Diode Array detector, a 20 µL injector and a computer integrating apparatus (EZ Start 7.4 software, Shimadzu Scientific Instruments, Inc., Columbia, MD, USA). Chromatographic separation was achieved on a reversed-phase column ACE^®^ EXCEL 5 C18-AMIDE (5 µm, 4.6 mm × 125 mm), a mobile phase consisted of methanol (A) acetate buffer 5 mM pH 6.5 (B). For separation the gradient method was developed as follows: A: B (0.5:99.5→0.01–3.00 min, 80:20→3.00–7.00 min; 80:20→7.00–22.00 min; 0.5:99.5→22–26 min). The flow rate was set at 1 mL/min, the UV wavelength range 200–700 nm and set 428, 310 and 250 nm to identification of MTR, CUR and GA, respectively. In these conditions the retention time of MTR, CUR and GA were 1.95, 12.42 and 15.68 min, respectively.

For CUR quantification, the calibration curve was performed in the concentration range of 0.0075–0.01 mg/mL. HPLC reports were highly reproducible and linearly related to concentration (R = 0.999).

### 2.9. Data Analysis

Data were expressed as mean ± SE. For the statistical analysis of the data, Student’s *t*-test has been applied with the minimum levels of significance with *p* < 0.05.

## 3. Results and Discussion

### 3.1. CUR-NLC Formulation and Characterization

In the pharmaceutical field, nano-encapsulation is highly exploited to promote the administration of substances with a low ability to cross biological membranes, using natural and/or synthetic materials to have a formulation with suitable chemical–physical characteristics. The low stability and solubility in a water solution of curcumin involve its delivery by NLCs [25].

Therefore, as components for lipid mixtures were chosen, long-chain fatty alcohol, such as 1-Hexadecanol, isopropyl palmitate, a liquid excipient able to solubilize lipophilic drugs, and 18-β glycyrrhetic acid (GA) with emulsifying properties. GA is a triterpenoid metabolite of glycyrrhizin, extracted from the dried roots and rhizomes of *Glycyrrhiza glabra* [36], studied for its anti-ulcer, antihepatotoxic, anti-microbial, anti-fungal, antitussive, anti-diabetic, anti-diuretic, skin whitening, cytoprotective and cytotoxic activities [37]. Moreover, it is well known as antioxidant tested against b-carotene destruction and LDL oxidation [38] and as anti-inflammatory agent, used for treating acute and chronic dermatitis reducing skin lesion sizes [39].

In order to prepare CUR-NLCs with high loading capacity and good stability is crucial the choose of a lipid mixture able to permit CUR solubilization and to confer to the whole mixture (lipids and CUR) a melting point similar to the physiological temperature of the oral cavity. Indeed, once applied on the mucosal target sites, NLC should undergo partitioning into the tissue and promote drug penetration.

Lipid screening study results (Table 1) showed how the ratios of high-melting and low-melting components affect the final melting point of the mixes.

Mixtures 1 and 2 had to be discarded for the low melting point, due to the high amount of IP, while Mixtures 3 and 4 presented melting points suitable for NLCs preparation.

In particular, CUR up to 2.5% w/w showed completely solubilized in Mixture 4, which appeared clear in the molten state.

Regarding the NLC preparation method, a homogenization followed by high-frequency sonication was performed. A hot pre-emulsion of melted lipid mixture and the aqueous emulsifier phase is obtained by hot homogenization, carried out at temperatures above the melting point of the lipids. A citrate buffer at pH 6.2 was chosen as an aqueous medium due to the pH-dependent instability of CUR showed as decomposition at a neutral or basic pH [40]. To reduce the particle size, sonication by a high-intensity ultrasonic probe was performed at the same temperature. Subsequently, the obtained hot nano-emulsion was treated by the second cycle of ultrasonication at 0 °C, which leads to the crystallization of the lipids and NLC formation. All the obtained dispersions appeared stable, homogeneous and easily to re-disperse.

Particle size, size distribution and Zeta potential are essential factors to value the stability of colloidal systems, showing significant effects on the final nanoparticles’ behavior [41].

Table 2 shows the DLS measurements of average particle size, particle size distribution and electrical charge surface of the nanoparticles expressed respectively as Z-average, PDI and Z-potential.

The surfactant amount is an important factor to determine the nanoparticle physicochemical characteristics due to its surface-active properties. To prepare NLCs with a smaller particle size, the concentration of surfactant could be increased in order to decrease the interfacial tension between lipid and aqueous phases. From Mixtures 3 and 4 were prepared CUR-NLCs with 0.5% or 1% of Tween 20, to evaluate any difference whether in particle size or system stability. The values were comparable and they clearly suggested that the lowest percentage of surfactant was sufficient to cover the surface of nanoparticles and prevent the lipid agglomeration during the homogenization process [42]. In particular, this stability may be enhanced by both the electrostatic repulsion between particles with the same charge and the stabilization and steric hindrance of Tween 20 chains. Although NLC containing 1% CUR showed smaller particle size and a good PDI value, the NLC from Mixture 4, showing suitable average particle size, the PDI value that indicates a narrow size distribution and Z-potential that reflects great physical stability of the dispersion, was preferred due to the higher CUR load. These results demonstrate that the amount of CUR loaded into the NLC plays a fundamental role in particle size.

The empty samples were also analyzed to understand the CUR contribution in system stability. The lack of CUR caused an increase in the ZP value, while the other parameters did not change significantly. However, this value suggests a low physical stability.

According to the last considerations, the formulations made by Mixture 4 and 0.5% Tween 20 were chosen for the next characterizations.

**Figure 1:**
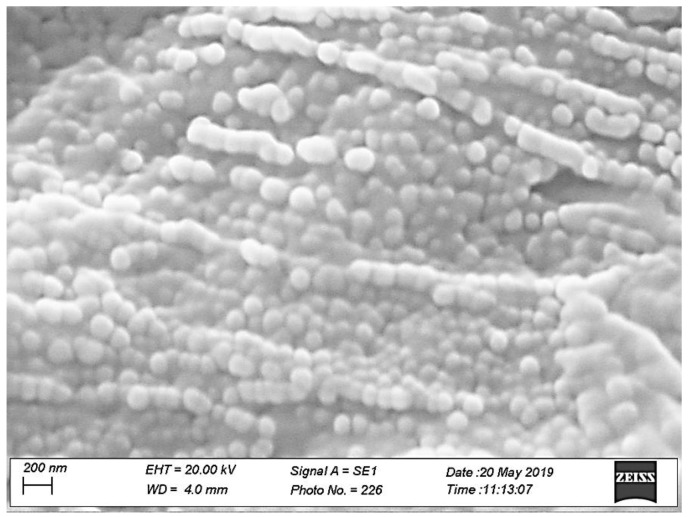
SEM morphology of CUR–NLC, showing the surface structure of the dried dispersion of CUR–NLC. Bar = 200 nm.

To obtain information concerning the surface characteristics and the morphology of CUR-NLC, SEM analysis was also performed and scanning electron micrographs are presented in Figure 1. NLCs show uniform spherical shapes with smooth surfaces, and their sizes was found evenly within the nanometer range. It is observable that the agglomeration of particles may be due to the lipid nature of carriers and the sample treatment prior the SEM analysis.

The drug recovery (DR) of CUR was calculated from the effective volume of fresh NLCs dispersion obtained (about 18 mL) with respect to the volume of buffer initially used (20 mL), where CUR resulted in 0.11 mg/mL, determining the 88% of DR. Furthermore, no degradation products were detected during CUR quantification by HPLC analyses.

EE and DL were indirectly determined, evaluating the amount of free CUR remaining left outside the nanoparticles by the dialysis and the ultrafiltration assays. In both the analyses no free CUR was found, so the EE was 100%. The DL calculated was of 2.2% (initial CUR 2.5%) that may be due to the loss of volume of the sample during the preparation process.

However, the high EE and DL values confirm the solubility of CUR in the chosen lipids as well as the suitable formulation design for CUR encapsulation.

### 3.2. Sponges Loaded with MTR and CUR-NLCs and Tablet Formulation and Characterization

Sponges were prepared entrapping the CUR-NLCs and MTR in a mucoadhesive hydrophilic polymeric matrix subsequently dried.

Natrosol^TM^, a nonionic, biocompatible, mucoadhesive, nontoxic polymer was chosen to prepare a hydrogel together with PVP-90 as mucoadhesive plasticizer and trehalose as cryoprotectant. These excipients were selected for the suitable lyophilization behavior and the acceptable physical appearance of the resulting products.

**Figure 2:**
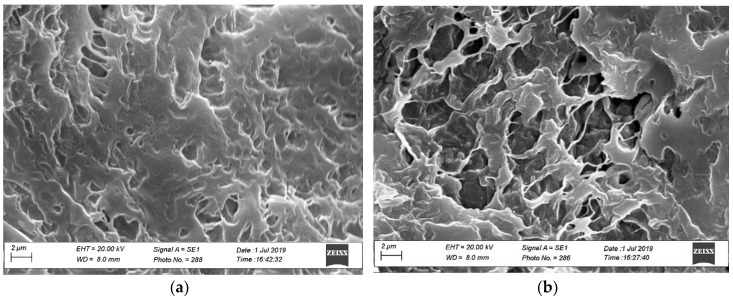
SEM of surface morphology and internal structure of a hydrophilic matrix (**a**) and hydrophilic matrix loaded with CUR-NLC and MTR (**b**). Bar = 2 μm.

The freeze-dried samples appeared soft, porous and friable. Besides the matrix morphology, the inner structure and surface topography were evaluated by SEM (Figure 2). The images showed an irregular structure with varying pore sizes and a high porosity that allows a more rapid entrance of water during dissolution that might mean an increased drug release rate. No drug crystal or aggregation of particles was visible in the photograph.

The homogeneous distribution of MTR and CUR into the lyophilized matrix was verified withdrawing portions of powder of the same batch and quantifying the drugs by UV-Vis analysis.

The results showed an average content of MTR and CUR of 9.57% and 0.25% (w/w), respectively.

The tablets have been designed to adhere and swell once applied on the mucosae, promoting an adequate penetration of both hydrophilic and hydrophobic actives. Indeed, they allow a precise location and adhesion on the target site, an extended retention time of formulation and an enhanced delivery in situ of actives.

Buccal tablets were prepared from the compression of aliquots of sponges obtained after the freeze-drying process. Tablets of 20 mg (A) and 200 mg (B) of sponge were made to perform different characterization experiments.

The reproducibility of the tablet’s preparation has been assessed measuring the average weight and thickness of both A and B ones. They were respectively 20 ± 0.3 mg, 2.34 ± 0.05 mm for Tablet A and 200 ± 1.2 mg 3.31 ± 0.05mm for Tablet B. All data are following the requirements of the Italian Pharmacopoeia (F.U. XII ed.) and confirm high product reproducibility.

After compression, they preserved the porous structure generated during the freeze-drying process, with the solvent removal. When the structure surrounding the liquid is rigid enough to prevent pore collapse, the pore structure is retained. So, the remaining space originally occupied by the solvent becomes pores in the scaffold. By investigation, the porosity of the tablets was calculated to be 96, 8%. This value was perfectly in accordance with the spongy matrix shown in the images obtained by SEM analyses.

**Figure 3:**
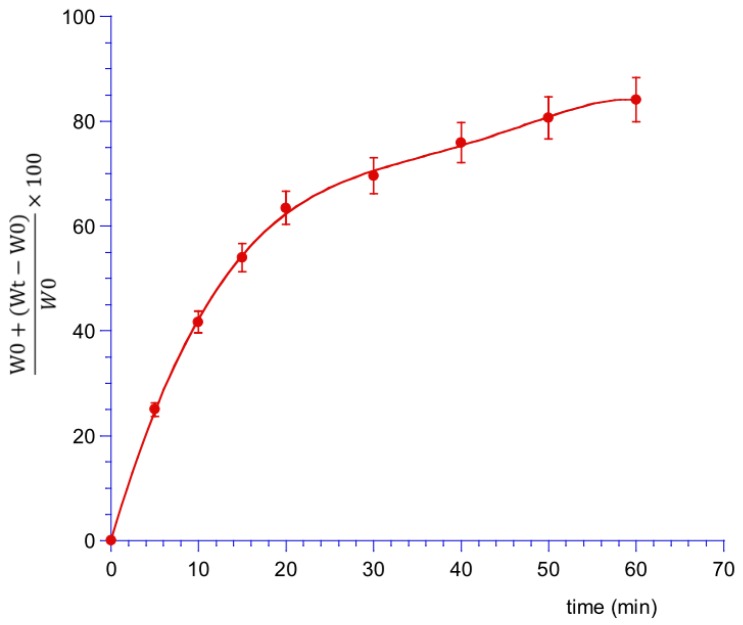
Swelling index measured as percent of weight increased vs. time. Values are presented as means ± SE (*n* = 9).

**Figure 4:**
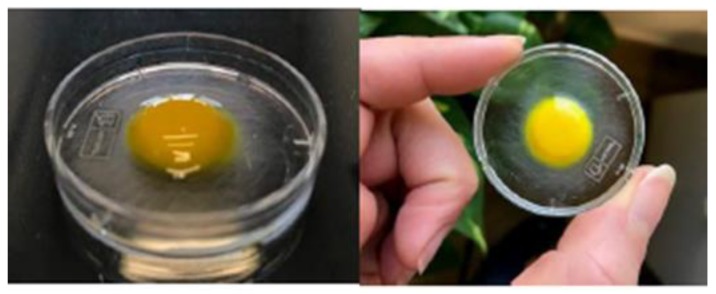
Tablet after 60 min of contact with simulated saliva.

For a suitable behavior and a sustained and controlled drug release, water penetration and tablet hydration capacity are critical properties here evaluated performing swelling tests as weight and optic assessments. Weight variations of the tablets are expressed as perceptual weight increase versus time, and they are reported in Figure 3. The data showed a rapid increase in weight in the first 20 min-interval, equal to 60% of the initial one, due to the absorption of saliva and the consequent swelling. After this time a much slower fluid absorption follows due to the formation of a viscous gel layer (Figure 4) that remains on the mucosa until a slow erosion phenomenon occurs.

**Figure 5:**
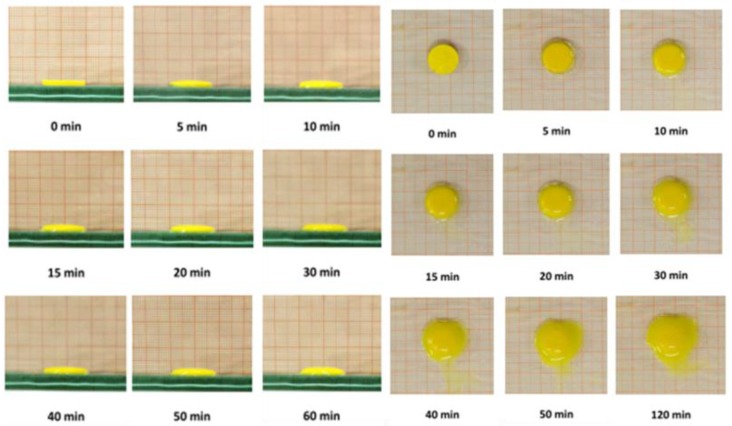
Timeline from 0 to 120 min of the plan and frontal swelling of the tablet.

The plan and frontal optical assessments (Figure 5) were carried out to measure the surface area of the oral mucosa, which is effectively covered by the tablet when dissolution occurs once it was applied on the target site. Both the frontal and plan photographs showed that the swelling of the tablet in the first 20 min was due to the main absorption of saliva that does not involve a large increase in volume and a partial loss of weight, and dissolution occur in 120 min.

### 3.3. Ex Vivo Mucoadhesion Strength Measurement

The mucoadhesive properties of buccal tablets influence the ability of the dosage form to be retained at the site of action, in intimate contact with the target site. For designing mucoadhesive tablets, polymers as polyacrylic acid and cellulose derivatives are often chosen for their suitable physical and chemical properties. Ex vivo mucoadhesion strength measurements were performed in triplicate by a modified two-armed physical balance, applying the tablets on fresh-cut porcine buccal mucosa. Variation in adhesive strength as a function of contact time was evaluated, and as is shown in Table 3, the mucoadhesive force increased with the increase of the contact time of formulation with mucin.

These suitable mucoadhesive properties are enhanced by the use of Natrosol and PVP-K90, polymers able to form addition bonds with mucins increasing the residence time of formulation on target mucosal site.

The release of drug molecules or nanoparticles from prepared tablets is necessary for their transport into and across the buccal membrane to exhibit therapeutic activity. However, the release of drugs from a swellable matrix depends on water diffusion into the matrix, polymer swelling and gel formation, then drug diffusion through the gel or its liberation from the gel [43].

**Figure 6:**
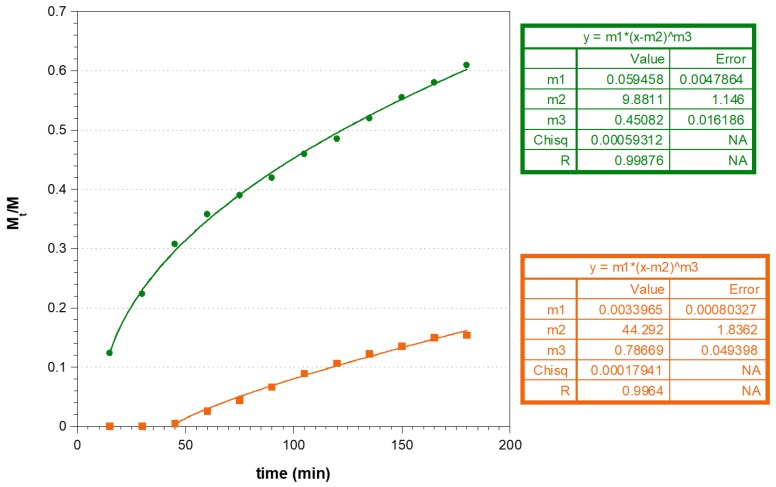
Plot of cumulative amount of MTR (●) and CUR (■) released from Tablet A and their fit with the modified Korsmeyer–Peppas model of MTR (--) and CUR (--).

Figure 6 analyses the dose fraction of CUR and MTR released from Tablet A at specific time points. The period of the drug release testing was kept for 3 h, as about 60% of MTR and 17% of CUR release were observed during this time.

Different physico-chemical phenomena are involved in the matrix tablet release mechanism. From the data, it is possible to highlight that there is a lag time in the release of CUR, probably because the matrix must initially absorb water and subsequently swell, then releasing the NLC containing CUR. This phenomenon is much less evident in the release profile of the MTR as it dissolves immediately in the fluid absorbed by the tablet and diffuses through it. By the fitting the mathematical models that are useful for comprehension of all the phenomena affecting drug release kinetics, the modified form of Korsmeyer–Peppas model, as described:

$$\frac{M_{t}}{M_{\infty }}=\;K{\left(t-l\right)}^{n}$$

where *M_t_* is the amount of drug released over time *t*, *M*_∞_ is the amount of drug contained in the dosage form at the beginning of the release process, *K* is the constant of incorporation of characteristics of the system, *n* is the exponent of release (related to the drug release mechanism and geometry of dosage form) in function of time *t* and *l* is the latency time, which marks the beginning of drug release from the system; the most appropriate to describe the behavior of our matrix tablet (Figure 6).

Experimental *n* values obtained by fitting can explain the complex phenomena that occur during MTR and CUR-NLC release from the tablet. Possibly, the MTR behavior follows a Fickian model (*n* = 0.45, for cylinders) and the release is governed primarily by diffusion. MTR is readily solubilized, and the solvent transport rate or diffusion is much greater than the process of polymeric chains relaxation. For CUR-NLC release, the value *n* = 0.77 indicates that the model is non-Fickian or anomalous transport, and the mechanism of drug release is governed by both diffusion and swelling. This could be attributed to the hydrophobicity or dimensions of the nanoparticles, so that their diffusion through the matrix is influenced by the swelling or relaxation of polymeric chains.

### 3.4. Ex Vivo Permeation and Penetration of CUR and MTR throughout Porcine Mucosa

The ability of the tablets to release CUR and MTR and the aptitude of actives to penetrate and/or permeate the membrane was evaluated using vertical Franz type diffusion cells and porcine buccal mucosa, a useful model to simulate human epithelium [34].

**Figure 7:**
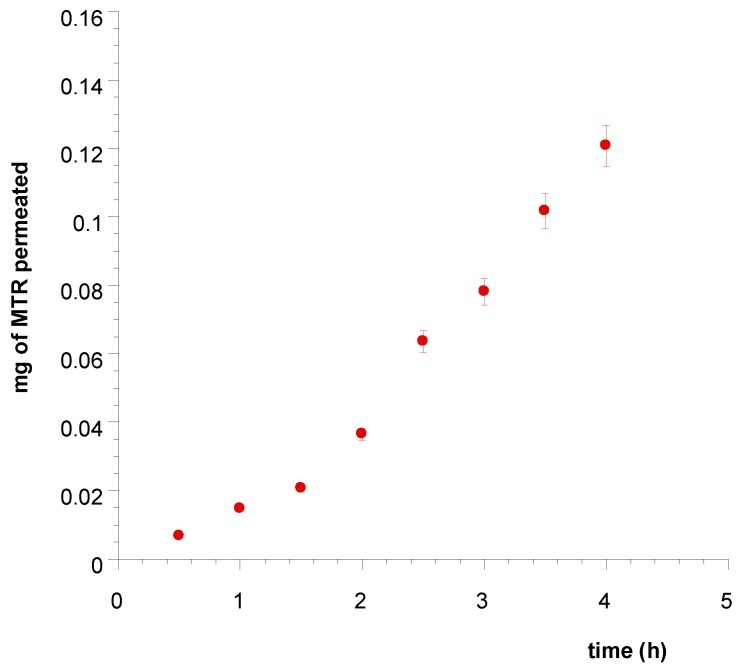
Plot of the cumulative amount of MTR permeated across porcine buccal mucosa vs. time from Tablet A soaked with simulated saliva. Values are presented as means ± SE (*n* = 12).

The spectrophotometric analyses demonstrate that just MTR permeates the buccal membrane, indicating its increasing concentration in the acceptor compartment. The drug movements from tablets to artificial plasma expressed as an accumulative amount of permeated MTR versus time, are showed in Figure 7.

**Figure 8:**
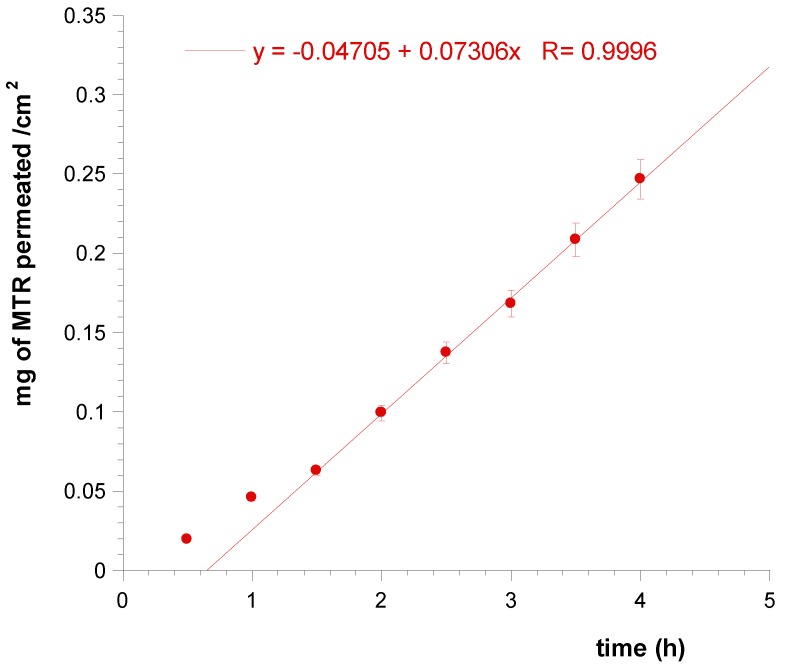
Linear fit at the steady state of MTR permeation per cm^2^ of porcine sublingual mucosa.

Tablets applied on porcine buccal mucosa for four hours produced massive input of MTR in the acceptor compartment and the extrapolated flux (Js) per unit area of MTR through the mucosal membrane at the steady state resulted 0.0731 mg/cm^2^ h (Figure 8). However, the experiments of drug permeation occurred from just one side of the tablet adhered on the mucosa, while in vivo conditions may imply that each side of tablets is in contact with the mucosa. So, a tablet of 1 cm^2^ produces a drug flux of about 0.146 mg/cm^2^h.

The residual CUR and MTR entrapped into the membrane were quantified by methanol extraction and analyzed respectively by HPLC and UV-Vis assays. After 4 h, the average amounts extracted were 0.0032 mg of CUR and e 0.0835 mg of MTR, that are respectively 6.31% and the 4.37% of the dose applied. These results demonstrate the ability of NLC to promote the penetration of CUR through the lipophilic domains of the mucosal membrane, where its accumulation can carry out its antioxidant activity. Moreover, results show that MTR is also able to penetrate and permeate the mucosal tissue, where it can perform a loco-regional antibacterial activity. The Ac parameter calculated for both the actives, 0.1006 and 0.0687 cm for CUR and MTR, respectively, demonstrated that the lipophilic molecule CUR possesses great tendency to penetrate lipophilic domains of membrane and accumulate into that. On the other hands, CUR is very scarcely soluble in aqueous fluids so that, without lipid nanocarriers, it cannot penetrate the tissue. By contrast, the MTR having a fair solubility in aqueous fluids, and due to its partition coefficient, can diffuse through the tissues and be distributed both in aqueous and lipophilic domains.

## 4. Conclusions

In this work, a novel buccal drug delivery system for the topical treatment of oral diseases related to both oxidative stress and bacterial overgrowth was designed. Nanostructured lipid carriers containing CUR have been formulated, characterized and successfully incorporated into a mucoadhesive hydrophilic sponge also containing MTR. The buccal tablets obtained by soft compression of sponge showed an appropriate porosity and swelling index as well as mucoadhesion strength. Ex vivo studies have shown that the tablets are able to release the actives promoting CUR penetration and MTR permeation. The obtained results encourage further studies about the possibility to use these matrix systems to deliver in situ antioxidant and antimicrobial agents to treat several diseases that affect oral mucosae. Finally, this matrix tablet was specifically designed to contrast oral infections, for which MTR is the drug of choice. However, this new DDS could be loaded with other drugs and potentially be useful for various pathologies affecting other mucous membranes (e.g., vaginal, rectal or nasal).

## Figures and Tables

**Table 1:** Lipid mixture compositions.

Sample	CUR %	GA %	HEXA %	IP %	Melting Point °C
Mixture 1	1	1	39.2	58.8	31 ± 2 °C
Mixture 2	1	1	58.8	39.2	40 ± 2 °C
Mixture 3	1	3	58	38	45 ± 2 °C
Mixture 4	2.5	3	56.7	37.8	48 ± 2 °C
Empty	0	3	57.3	38.2	42 ± 2 °C

**Table 2:** Results of dynamic light scattering (DLS) and Z-Potential measurements.

.	Tween 20%	Z-Average (nm)	PDI	Z-Potential (mV)
**Mixture 3**	0.5	100	0.242	−24.6
	1	100	0.274	−24.4
**Mixture 4**	0.5	121.6	0.235	−37.4
	1	121.8	0.272	−33.2
**Empty**	0.5	129.4	0.216	−8.4

**Table 3:** Force of adhesion and detachment force of the tablet after different contact time on the porcine buccal mucosa (*n* = 3).

Contact Time (min)	Force of Adhesion (N)	Detachment Force (N/m^2^)
**5**	0.064	486.81
**10**	0.073	549.50
**15**	0.100	752.35
**20**	0.146	1095.33
